# CRISPR/Cas9 mediated chicken Stra8 gene knockout and inhibition of male germ cell differentiation

**DOI:** 10.1371/journal.pone.0172207

**Published:** 2017-02-24

**Authors:** Yani Zhang, Yingjie Wang, Qisheng Zuo, Dong Li, Wenhui Zhang, Fei Wang, Yanqin Ji, Jing Jin, Zhenyu Lu, Man Wang, Chen Zhang, Bichun Li

**Affiliations:** 1 College of Animal Science and Technology, Yangzhou University, Yangzhou, Jiangsu province, P.R. China; 2 Key Laboratory for Animal Genetics, Breeding, Reproduction, and Molecular Design of Jiangsu Province, Yangzhou, Jiangsu province, P.R. China; Qingdao Agricultural University, CHINA

## Abstract

An efficient genome editing approach had been established to construct the stable transgenic cell lines in the domestic chicken (Gallus gallus domesticus) at present. Our objectives were to investigate gene function in the differentiation process of chicken embryonic stem cells (ESCs) into spermatogonial stem cells(SSCs). Three guides RNA (gRNAs) were designed to knockout the Stra8 gene, and knockout efficiency was evaluated in domestic chicken cells using cleavage activity of in vitro transcription of gRNA, Luciferase-SSA assay, T7 endonuclease I assay(T7E1) and TA clone sequence. In addition, the Cas9/gRNA plasmid was transfected into ESCs to confirm the function of Stra8. SSA assay results showed that luciferase activity of the vector expressing gRNA-1 and gRNA- 2 was higher than that of gRNA-3. TA clone sequencing showed that the knockdown efficiency was 25% (10/40) in DF-1 cells, the knockdown efficiency was 23% (9/40) in chicken ESCs. T7E1 assay indicated that there were cleavage activity for three individuals, and the knockdown efficiency was 12% (3/25). Cell morphology, qRT-PCR, immunostaining and FCS indicated that Cas9/gRNA not only resulted in the knockout of Stra8 gene, but also suggested that the generation of SSCs was blocked by the Stra8 gene knockdown in vitro. Taken together, our results indicate that the CRISPR/Cas9 system could mediate stable Stra8 gene knockdown in domestic chicken’s cells and inhibit ECSs differentiation into SSCs.

## Introduction

Stra8 (stimulated by retinoic acid gene 8) was a gene regulated by retinoic acid (RA), which was a specific expressed gene from mitosis to meiosis for mammalian germ cell. Previous investigator showed that Stra8 was specifically expressed in embryonic and postnatal gonads[1]; meanwhile, it was expressed in cytoplasm and nuclei of germ cell[2]. When the cells were in different functional state, Stra8 could shuttle between the cytoplasm and the nucleus to play its function. Stra8 gene knockout mice led to male sterility, and accompanied some abnormal phenotypes related to meiotic cell cycle, chromatin condensation, homologous recombination, DNA repair and cell apoptosis[3]. Although Stra8 gene was essential in the process of human and mouse spermatogenesis, the molecular regulatory network of Stra8 in spermatogenesis has not been elucidated.

Chicken as the classic model of developmental biology, because of its unique embryonic development process, a large number of ESCs could be gotten from blastocysts and a variety of in vitro operation, such as micro-injection, could be conducted in any period of development; Chicken also was used to produce chimeras or researched on embryonic development, cell migration, formation of tissues and organs. Therefore, more and more research was developed in domestic chicken and the explored specific mechanisms of some genes during embryonic stem cells into male germ cells.

Gene editing was an important method to study the function of genes [4–7], which had become increasingly matured in gene knock-in and knock-out for mammals. Among the entire gene editing technology, CRISPR/Cas gene knockout technology was widely used and mediated the stable gene knockout. Compared with ZFN and TALEN technology, the targeting efficiency of CRISPR/Cas (up to 80%) was relatively higher than both of them [8], meanwhile, it was very flexible and convenient to design the target site. So, based on the popular usage of CRISPR/Cas and its unique merits, the purpose of this study was to construct the over-expression vector and CRISPR/Cas mediated gene knockout vector of Stra8 gene, chicken embryonic stem cells were transfected by both of them separately, the cell’s morphology was observed, the variation trend of germ cell specific gene markers, flow cytometry screening was conducted to analyze the regulation network of Stra8 from chicken embryonic stem cells differentiation into germ cells. The results of this study will not only provide theoretical basis for the application of CRISPR/Cas technology in the domestic, but also provide a model for the molecular regulation mechanism of Stra8 gene in vitro.

## Materials and methods

### Ethics statement

All the procedures involving animals and their care were conformed to the U.S. National Institutes of Health Guidelines (NIH Pub. No. 85–23, revised 1996). The whole experiments procedures were approved by the Ethics Committee of Yangzhou University for Laboratory and Experimental Animals and the Institutional Animal Care and Use Committee of Yangzhou University. The Rugao yellow chickens used in this study were provided by the Institute of Poultry Science, Chinese Academy of Agriculture Sciences.

About 300 fresh fertilized eggs were cleaned by 0.1% of Benzalkonium bromide and 75% alcohol, then embryonic stem cells(ESCs) were collected. 90 fresh fertilized eggs were hatched in Sterile incubator from wansheng WSC company with the incubation conditions set as: temperature 38.5℃, humidity 60, and egg turning once every 1 hour by the angle of 90 degrees. we sacrifice the hatched chicken embryos by putting them into aether for 1 to 2 minutes Then the tissues were harvested from the chicken embryos with no breath and nerve reflex. The nerves won’t be excited, and all the procedures were carried out in painless conditions.

### Materials

The chicken embryo fibroblast cell line, DF-1 were purchased from ATCC. The Luciferase-SSA vector was constructed by our laboratory; VK001-08 (gRNA/Cas9 expression plasmid, U6 promoter gRNA expression, CMV promoter Cas9 protein expression) was purchased from Beijing viewsolid biotech co.ltd.

### Methods

#### Stra8 gene cloning and gRNA design

To clone Stra8 gene, we designed the following primers based on the mRNA sequence obtained from the NCBI database (ID: XM_416179): forward, 5' CCCAAGCTTATGCAAGAATGTGAAAAAC3'; reverse, 5' CCGGAATTCTTATAAATCTTCATCATCA3'. We identified the coding sequence region based on the protospacer adjacent motif (NGG or GGN) using the initial 19–21 bp for gRNA design. To avoid off-target effects, the entire genome was searched for potential off-target sites. The gRNA sequences were shown in Table 1.

**Table 1:** Nucleotide sequence of gRNA to target site.

Name	Sequence of gRNA	PAM
gRNA-1	AATGTGAAAAACAAACAA	TGG
gRNA-2	TTCAAATTCCACCAGATCTGG	TGG
gRNA-3	GAGCTACTGGGAGAGCA	TGG

Oligo primers were set according to the selected gRNA, the primers sequence was 5 'AAACACCG-gRNA-1, 5'CTCTAAAAC-gRNA-1-anti; 5'AAACACCG-gRNA-2, 5'CTCTAAAAC-gRNA-2-anti; 5 'AAACACCG-gRNA-3, 5' CTCTAAAAC-gRNA-3-anti. Step 1: The sense strand and antisense strand was annealed to form a double stranded oligo; annealing system was 1μL of each sense strand and antisense strand, 1 μL Solution, 3 μL H_2_O. Annealing procedures: 95℃ for 5min, natural cooling to 16℃, 16℃ for 10 min. Step 2: oligo dipolymer were inserted into vector; the reaction system: 1μL Cas9/gRNA vector, 2 μL oligo dipolymer, 7 μL H_2_O. The reaction procedure was 25℃ standing for 5 min. The 5~10 μL final product obtained in step 2 was added into 50 uL DH5α competent cells thawed freshly, fully mixed, put on ice for 30 min, 42℃ heat shock on ice for 90 seconds, standing for 2 min, and cultured in LB solid medium supplemented with 0.1% ampicillin for 12–16 h. Positive clones were picked out and cultured in LB liquid medium supplemented with 0.1% ampicillin for 12–16 h, the recombinant plasmids were extracted using Plasmid Minispin Kit (DingGuo Biotech Company, China). Plasmid containing the right insertion was then sequenced by Sangon Biotech Company (Shanghai, China). The sequence primer was TGAGCGTCGATTTTTGTGATGCTCGTCAG.

#### Cell culture and transfection

DF-1 cells were chicken embryo fibroblast cell line, were cultured in Dulbecco’s Modified Eagle’s medium (high glucose, Sigma) supplemented with 15% (v/v) fetal bovine serum (Invitrogen), 100 IU/ml penicillin and 100 μg/ml streptomycin. All cultures were maintained in a 5% CO_2_ humidified atmosphere at 37°C and passaged every 2–3 days. Cells were grown to approximately 60% confluency for transfection purposes. Fugene were used as the transfection reagent, the ratio between Fugene and plasmid was 3:1. Flow cytometry and cell sorting was conducted after 48 hours transfection to collect the positive cells.

#### Luciferase-SSA assay, T7 endonuclease I assay(T7E1) and TA clone sequence

For construction of the single-strand annealing (SSA) luciferase reporter, we inserted a terminator sequence followed by the gRNA target sequence into a plasmid encoding the luciferase gene. The SSA luciferase reporter was co-transfected with the CRISPR/gRNA vector and a Renilla luciferase reporter (internal control). An empty plasmid was used as a negative control. At 48 h after transfection, luciferase SSA activity was detected by Synergy™ 2 Multi-Mode Microplate Reader (Biotech, Vermont, USA). To test luciferase signal, we inserted terminator and target site into the Luciferase vector to co-infect the CRISPR/gRNA vector, the newly constructed luciferase reporter gene and the internal control renilla plasmid. Transfected void vector was set as control. The target site is located after the terminator. After 48 h transfection, GFP positive cells were screened out using flow cytometry from the secondary generation cells. Genomic DNA of positive GFP cells was extracted for T7E1 assay. The following primers were used to do polymerase chain reaction: F: 5' GTATTTCCTTTGCTTCCTATGC 3' and R5' TGATACCACACTGGGATTCCAT 3'. About 511bp fragment including the target site was cloned and confirmed by the T7E1 enzyme. TA clones (*n* = 30) was picked up for sequencing and the efficiency of CRISPR/Cas9 gene knockout vector was calculated. Gene knockout efficiency = counts of mutated bacterial community/total sequenced bacterial community *100%.

#### In vitro transcription of gRNA and cleavage activity

The fragment of gRNA was used as template, T7-gRNA was used to do amplification to obtain PCR product of 120bp, and PCR product was purified and used as DNA template for the subsequent in vitro transcription. The target gRNA (PAM sequences) was inserted into the PCR products through bridging PCR. In vitro transcription processes were shown in vitro transcription kit (Roche kit). Follows was PCR reaction conditions: gRNA plasmid 10ng, T7-gRNA-FPg (10μM) 1.5μL, gRNA-RP (μM) 1.5μL, 2×Pfu Mix 2μL, double distilled water up to 50μL. PCR amplification program, Step1: 95℃ 3 min; Step 2: 94℃ 30 sec, 58℃ 30 sec, 72℃ 30 sec for 35 cycles; Step 3: 72℃ 10 min; 16℃ 10 min. Meanwhile, PCR products of standard gRNA1 (g1) and gRNA2 (g2) was prepared. The primer sequence of T7-gRNA-F was: TAATACGACTCACTATAG-gRNA-GTTTTAGAGCTAGAAATAGC and gRNA-R: AGCACCGACTCGGTGCCACTT. In vitro enzyme digestion conditions were shown in Table 2.

**Table 2:** In vitro enzyme digestion conditions.

	1	2	3
Cas9	1U	1U	1U
10X Cas9 buffer	2 μL	2 μL	2 μL
gRNA	50 ng (gRNA)	50 ng (Positive)	50 ng (Negative)
ddH_2_O	X μL	X μL	X μL
dsDNA	50 ng	50 ng	50 ng
Total	20 μL	20 μL	20 μL

#### In vitro induction experiment

Isolation and culture of chicken ESCs: Fresh fertilized eggs were disinfected by 1‰ Benzalkonium Bromide and 75% alcohol. The blunt end of egg was broken by the tweezer and eggwhite was removed, then blastoderm cells at stage X were collected by spoon method[9] in tissue culture dishes and rinsed with Ca2+ and Mg2+ free phosphate-buffered saline (PBS-CMF) to remove the yolks and vitelline membrane. After washing with PBS, ESCs were transferred into fresh tissue culture dishes containing PBS with 0.25% trypsin and 0.04% ethylenediaminetetraacetic acid (EDTA). Followed was digestion at 37.0℃ for 5~8 min, the dissociated cells were collected by centrifugation at 1000 r/min for 8~10 min and suspended, then ESCs were maintained in a 5% CO2 humidified atmosphere at 37.0℃ with cytokine culture medium (DMEM (Sodium pyruvate, L- glutamine) +10% FBS+1% non-essential amino acid +5.5×10-5mol/L β- ethyl alcohol +2% chicken serum+10 ng/mL bFGF +0.1 ng/mL LIF+5 ng/mL SCF).

In vitro induction: The third-generation ESCs were cultured onto a 24-well plate with a density of 10^5^ cells per well, the overexpression or CRISP/Cas9 mediated knockout vector of Stra8 was transfected, respectively; meanwhile, the corresponding control vector was transfected as the reference. The medium was changed for DMEM (Sodium pyruvate, L- glutamine) +10% FBS+RA (10^-5^mol/L) after 24h transfection. The cells culture media was changed every two days, the morphological changes of the cells were observed by fluorescence inverted microscope. During the induction process, the sample was collected every 2 days, western blotting and qRT-PCR was conducted to confirm the expression of NANOG, Sox2, C-kit, Cvh, integrinα6 and integrinβ1, indirect immunofluorescence assay was used to detect the expression of germ cell specific genes after induction.

#### Microinjection of the Cas9/gRNA plasmid into chicken embryos

The polyethylenimine (PEI)-encapsulated CRISPR/Cas9 vector was injected into chicken embryos, which were then sealed with paraffin and incubated at 38.5℃ for 4.5 d. DNA was extracted for analysis using the T7EI assay.

#### Quantitative Real-Time PCR (qRT-PCR)

The cells were collected at 0 day, 4 days (PGC-like cell appear in vitro), 10 days (SSC-like cell appear in vitro) and RNA was extracted using the RNeasy kit(TIANGEN, Beijing, China) and reverse transcribed to cDNA.The primers used for qRT-PCR were listed in Table 3. Assays were conducted using the SuperReal PreMix Color (SYBR Green) (FP215, TIANGEN, Beijing, China) and performed on an ABI two-step RT-PCR system (Applied Biosystems 7500, USA) with diluted first-strand cDNA according to the manufacturer’s instructions. For each reaction, 0.1 ng of totalRNA was used as input. The qRT-PCR reaction mixtures included 1 μL of cDNA, 10μL of 2×SuperReal Color PreMix, 0.6 μL of the forward and reverse primers (10 μmol/L), 0.4 μL of 50×ROX Reference Dye, and RNase-free ddH_2_O in a total reaction mixture volume of 20 μL. The mixtures were put into a clear tube (0.2 mL thin wall, Axygen, USA). The reactions were comprised of 40 cycles of the following program: 95°C for 15 min, followed by 9500B0030C for 10 s, 60°C for 30 s, and 72°C for 32 s. The β-actin gene (house-keeping genes) was served as an internal reference gene, and all reactions were performed in triplicate. Gene expression levels were calculated using the 2^-ΔΔCT^ method.

**Table 3:** Primer information for qRT-PCR.

Gene	Primers for qRT-PCR	Tm(°C)	Size(bp)
*Nanog*	F: TGGTTTCAGAACCAACGAATGAAG	64	180
	R: TGCACTGGTCACAGCCTGAAG		
*Sox2*	F: GAAGATGCACAACTCGGAGATCAG	64	100
	R: GAGCCGTTTGGCTTCGTCA		
*C-kit*	F: GCGAACTTCACCTTACCCGATTA	64	150
	R: TGTCATTGCCGAGCATATCCA		
*Cvh*	F: TGTCTTGAAGGCCTCGTTTG	61	138
	R: CATATCCTTGGCAGGTTGTTGA		
*integrinα6*	F: TGTTTGTGGGGACCAGATTG	53	120
	R: CCAGGTGACATTTCCCATCA		
*integrinβ1*	F: GAAACCCGGGATATCATTGG	53	140
	R: CAGCAACACCTTGCTGACAG		

#### Immunofluorescence

Cell suspensions were incubated on poly-L-lysine-coated slides to adhere and fixed for 10 min in 4% paraformaldehyde. After 1 h of blocking in 10% bovine serum albumin (BSA) in PBS, antibodies against Integrinα6 (ab20142, Abcam, Shanghai, China) were added at a 1:200 dilution in 10% BSA in PBS and incubated overnight at 4°C. After washing three times with 0.05% Tween-20 in PBS, FITC-labeled secondary antibodies (Biosynthesis Biotechnology Co., Ltd.) were added at a 1:100 dilution for 2 h. After washing again with 10% BSA-PBS, the cells were observed under an inverted fluorescence microscope.

#### FACS

Cell suspension induced of 10 days were collected in 1.5 mL centrifuge tube, 1000 g centrifugal for 5 min, abandon the supernatant, add the integrinα6 (1:200) and integrinβ1 antibody, 4℃ for 1~2 h incubation, centrifugal supernatant. Cells were washed twice with precooling PBS, 500 uL precooling PBS was added for FACS analysis.

### Data analysis

SPSS19.0 software was used to carry out the t-test analysis (*P< 0*.*05* for significant differences, *P<0*.*01* for highly significant differences). EXCEL2003 was used to generate figures.

## Results

### CRISPR/Cas9 vector was constructed completely

**Fig 1:**
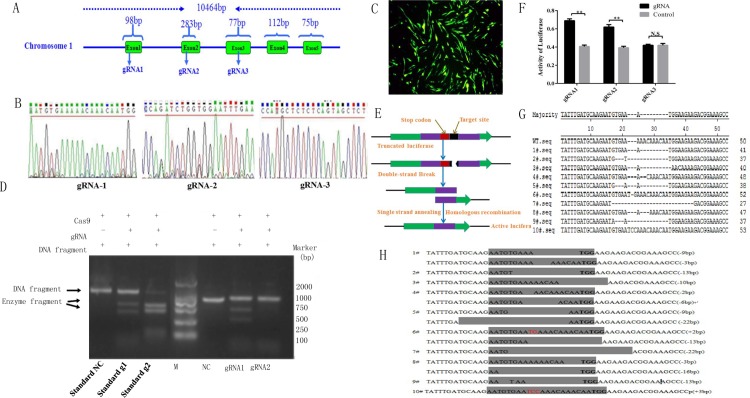
Cloning of Stra8 gene and construction of cas9/gRNA vector. A: The schematic diagram of Stra8 gene and the target site position. Three target sites were designed basing on the sequence at the exon 1, exon2 and exon3. B: Sequencing results after an avian derived U6 promoter was inserted into the vector. Sequencing results showed that gRNA1, gRNA2 and gRNA3 have been connected to the CRISPR/Cas9 vector completely. C: The Cas9/gRNA knockout vector was transfected into DF-1. D: The cleavage activity of Cas9/gRNA in vitro. SSA activity of the standard g1 and g2 were 3 and 10, respectively, SSA activity of gRNA1 and gRNA2 was between standard g1 and g2, the cleavage efficiency was 40%-50%.E: Flowchart for the SSA activity assay. F: The result of SSA showed that cas9/gRNA1 and cas9/gRNA1 has the knockdown activity. G: Results of the T7EI assay show a clear band at approximately 250 bp and gene knockout. H: Alignment of TA clone sequences. K: TA clone sequencing of monoclonal cells shows homozygous mutations in #1, #4, #5, #6 and #8, and heterozygous mutationsin the other cell lines.

The cDNA obtained from the chicken spermatogonial stem cells were used as template, primers were designed according to the sequence of Stra8 CDS published by NCBI database, and the whole gene sequence was 672 bp. Three target sites were designed basing on the sequence at the exon 1, exon2 and exon3 (Fig 1A), oligo primers aimed at the target site were designed too, and then annealing was conducted between oligo primers and target site. The target site sequence of gRNA was inserted into the CRISPR/Cas9 vector, which expresses Cas9 protein and gRNA simultaneously using the T7 and avian-derived U6 promoters, respectively. To facilitate cell screening, this plasmid expresses the puromycin resistance gene and GFP. Sequencing results showed that gRNA1, gRNA2 and gRNA3 have been connected to the carrier completely (Fig 1B).

### CRISPR/Cas9-mediated Stra8 gene deficiency in chicken DF-1

To determine whether CRISPR/Cas9 gene editing technology could knockout Stra8 genes in the cells derived from domestic chicken, the knockout vector and Luciferase-SSA reporter vector as well as donor vector were transfected into DF-1 cells (Fig 1C) to confirm the cleavage activity of Cas9/gRNA, gRNA1 and gRNA2. Cas9/gRNA, gRNA1 and gRNA2 was transcribed in vitro and connected into the PCR product to do enzyme digestion experiment, the results showed that the digestion efficiency of gRNA was between standard g1 and g2, the cleavage efficiency was 40%-50%(Fig 1D), suggesting that Cas9/gRNA had the higher knockdown activity. Meanwhile, SSA assay was used to further make sure the knockout efficiency (Fig 1E), the highest luciferase activity was shown by gRNA1 and gRNA2(Fig 1F). Then gRNA1 was transfected into DF-1 and genomic DNA was extracted after 48h transfection, PCR was conducted to amplify the specific fragment (511bp) containing the target site. T7E1 results showed the cleavage products for gRNA1(Fig 1G). TA clone sequencing showed that the knockdown efficiency was 25% (10/40) (Fig 1H). Subsequently, the limiting dilution method was used to obtain more than 10 monoclonal cell lines and DNA was extracted from the cell lines to do PCR. TA clone sequence results revealed that there were homozygous mutations in four cell lines and six heterozygous mutations in six cell lines(Fig 1K).

### CRISPR/Cas9-mediated Stra8 gene deficiency in chicken ESCs

**Fig 2:**
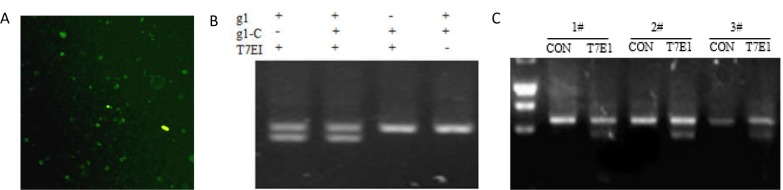
CRISPR/Cas9-mediated gene deficiency in chicken embryos. A: Effect of CRISPR/Cas9 plasmid transfection into chicken ESCs. B: Results of the T7EI assay show a clear band at approximately 250 bp in genomic DNA from ESCs. C: Results of the T7EI assay show a clear band at approximately 250 bp in genomic DNA from three individuals.

To further derermine whether CRISPR/Cas9 gene editing can knockdown genes in the stem cells derived from the domestic chicken, the purified second-generation ESCs was teansfected with Cas9/gRNA-1(Fig 2A). The genomic DNA was extracted after 48 h transfection, and a approximate 512bp fragement including the targeting site was cloned, then treated with T7E1, the knockdown efficiency was 23% (9/40) (Fig 2B), which suggested that CRISPR/Cas system could effectively knockdown gene in chicken ESCs.

### CRISPR/Cas9-mediated Stra8 gene deficiency in chicken and embryos

To determine whether the CRISPR/Cas9 editing technology could knockdown genes in chicken embryos, the Cas9/gRNA-1 vector was encapsulated by PEI. T7E1 assay indicated that there were cleavage activity for three individuals, and the knockdown efficiency was 12%(3/25) (Fig 2C).

### Stra8 knockdown mediated by Cas9/gRNA-1 inhibited the differentiation of ESC into male germ cells in vitro

**Fig 3:**
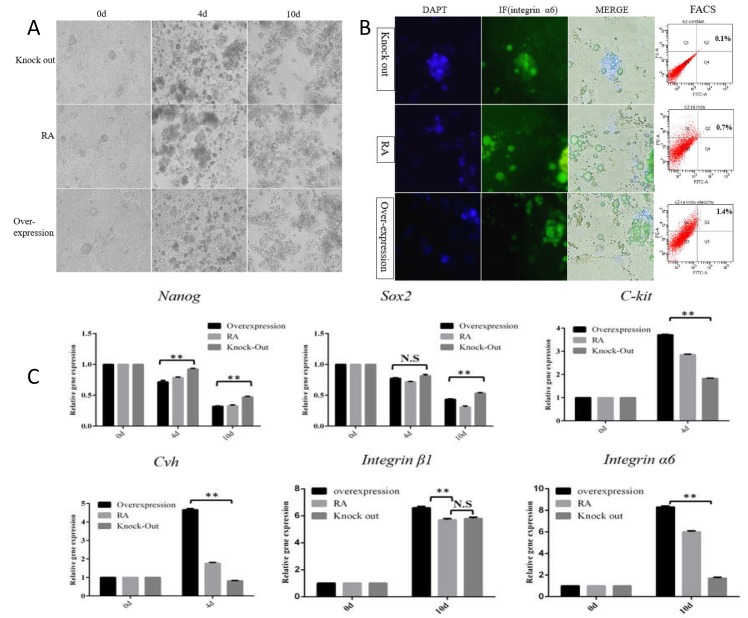
Stra8 knockdown mediated by Cas9/gRNA-1 inhibited the differentiation of ESC into male germ cells in vitro. A: Cell morphological changes during the induction of Stra8 gene knockout or overexpression. The overexpression or CRISP/Cas9 mediated Stra8 was transfected, respectively; meanwhile, the corresponding control vector was transfected too. B: Indirect immunofluorescence and flow cytometry results after 10 days of induction of cells (400×). Indirect immunofluorescence staining results of the SSC-specific proteins integrin α6 (green) and DAPI staining (blue), respectively. There were more SSC-like cells in RA induction group and Stra8 overexpression group. Cell suspension induced of 10 days marked by integrin α6 and integrin β1and FACS analysis was conducted. There were more SSC-like cells in RA induction group and Stra8 overexpression group. C: The trend change of germ cell specific mark gene during the induction of Stra8 gene knockout or overexpression. The cells were collected at 0 day, 4 day and10 day, RNA was extracted using the RNeasy kit (Qiagen) and reverse transcribed to cDNA. There was a significant upward trend for C-kit, Cvh, integrin α6 and integrin β1 expression in RA induction group and RA+Stra8 overexpression group, but no significant change in RA+Stra8 knockdown group, pluripotent gene Nanog and Sox2 decreased with the three induction group, suggesting that the differentiation of ESCs into SSCs was blocked by Stra8 deficiency.

The Stra8 knockdown vector mediated by CRISPR/Cas9 or over-expression vector was transfected into the third generation of ESC, which was cultured into the medium within RA induction. More embryoid bodies and germ cells appeared in RA induction group and RA+ Stra8 overexpression group, but it did not show for RA+ Stra8 knockdown group (Fig 3A). QPCR results showed that the expression of germ cell specific marker gene was significantly expressed in RA induction group and RA+ Stra8 overexpression group, but no significant change in RA+ Stra8 knockout group (Fig 3C).There was a significant upward trend for C-kit, Cvh, integrin α6 and integrin β1 expression in RA induction group and RA+Stra8 overexpression group, but no significant change in RA+Stra8 knockdown group, pluripotent gene Nanog and Sox2 decreased with the three induction group, suggesting that the differentiation of ESCs into SSCs was blocked by Stra8 deficiency (Fig 3C). Indirect immunofluorescence and flow cytometry showed that the positive cells number with the expression of integrinα6 was decreased greatly in RA+ Stra8 knockout group (Fig 3B), however, the cell number detected by FCS was increased in RA induction group and RA+ Stra8 overexpression group, indicating the generation of SSCs was blocked by the Stra8 gene knockdown in vitro.

### Stra8 plays an importmant role in SSCs formation during chicken embryo development in vivo

**Fig 4:**
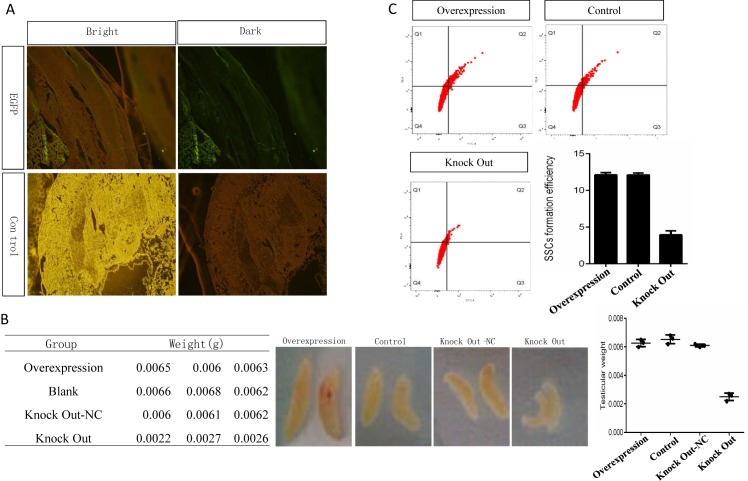
Stra8 knockdown mediated by Cas9/gRNA-1 inhibited the differentiation of ESC into male germ cells *in vitro*. A: Expression of the vector in chicken embryos, as assessed by frozen section observation. B: The results of testis weight showed that Stra8 knockdown could block the testis development during normal chicken embryo development. C: We separated SSCs from Stra8-overexpression and Stra8-knock down group after 18.5 d hathing, and marked with integrinα6 and integrinβ1. FCS results showed that there were less integrinα6+ and integrinβ1+ in Stra8-knock down group, further displaying that Stra8-knockdown can effectively suppress the formation of SSC.

To determine role of Stra8 in SSC formation during chicken embryo development, the PEI-encapsulated vector containing Stra8-knockdown vector was injected into chicken embryos, EGFP could be expressed after 4.5d hatching (Fig 4A). There was significant difference for the testis development after 18 d hatching between Stra8-overexpression and Stra8-knock down group (Fig 4B). We separated SSCs from Stra8-overexpression and Stra8-knock down group after 18.5 d hathing, respectively; And marked with integrinα6 and integrinβ1, then FCS was conducted. The results showed that there were less integrinα6^+^ and integrinβ1^+^ in Stra8-knock down group(Fig 4C).These results further displayed that knockdown of Stra8 can effectively suppress the formation of SSC.

## Discussion

It was very necessary to conduct gene knockout and permit the investigators to study gene function. Investigators had adopted natural DNA repair mechanisms to achieve site-directed modification of target genes; but since its low efficiency and poor repeatability is. Therefore, the simpler and more effective approaches were found to get gene knockout/knock-in, such as engineered endonuclease techniques. Although ZFN[10] and TALEN[11] has been widely used as the tools for gene function capture, the experimental procedures and construction design are complex. With the appearing of CRISPR/Cas9, ZFN and TALEN technologies was replacing quickly since CRISPR/Cas9 was simpler and more effieiency[12]. As the CRISPR/Cas9 was found, it has been widely used in animal cell level or individual level. For examlpe, stable knockout cell line has been produced in HEK293 cells and induced pluripotent cells produce stable knockdown cell lines, knockdown animals models had been achieved for mouse, rat and zebrafish by the microinjection method, the offspring was obtained successfully. Jinek et al. [10] and Sternberg et al.[11] had used CRISPR/Cas9 technique to produce DNA double-strand breaks, suggesting that the CRISPR/Cas9 system was possible to finish gene editing in humans. Sommer et al.[13] did a modification for the Cas9 system and finally achieved gene knockout in human HEK293 and K562 cells, the knockout efficiencies was 10~25% in HEK293 cells and 8~13% in K562 cells. Cong et al.[14] knocked out the EMX1 and PVALB genes in HEK293 cells successfully, and the Th gene in mouse Neuro 2A cells, the entire knockout was mediated by the CRISPR/Cas9 system. Cho et al. (2014) demonstrated that the gene knockout efficiency was improved in a co-transfection system with the increasing of gRNA concentration; the knockout efficiency was close to 33%. Cong et al. [15] demonstrated higher knockout efficiency was found when the gRNA’s structure was similar to the crRNA: tracrRNA complex. However, although Zhang et al. [16] reported that the CRISPR/Cas9 system was suitable for editing gene in any cell types, the more study about this technique just focused on the mammalian cells. Recently, there were two nice published paper using chicken cell lines. One of them was found by Veron and coworkers[17], CRISPR gRNA plasmids was directly to against the PAX7 transcription factor; another one was Bai and his coworkers[18], they edited the PPAR-g, ATP synthase epsilon subunit(ATP5E), and ovalbumin (OVA) genes in chicken DF-1 cells using CRISPR strategies. However, all these studies show only that the CRISPR/Cas9 can knockdown the gene in DF-1, but not does the function study for the deletion genes. For that reason, in order to verify whether the Cas9/gRNA system can achieve a precise gene knockout in poultry, the chicken embryo fibroblast cell line DF-1 was used as the experimental material, Stra8 gene mediated by Cas9/gRNA system in poultry was confirmed. The results showed that the Cas9/gRNA system could mediate Stra8 gene knockout in chicken DF-1 cells, and the knockdown efficiency was about 26%~27%, which was consistent with Zuo’ results, indicating Cas9/gRNA system can be stably used in chicken cells. Although a large number of studies showed that gene knockdown efficiency mediated by Cas9/gRNA could reach about 40%~80% in mammalian animal and plant, however, the cell quality and matured technology had become the important factors for the knockout efficiency since it was the first time to do Stra8 gene knockdown in chicken. But the results presented in this research could be enough to explain that Cas9/gRNA was able to get gene knockout in chicken, which not only opened up a new field for the application of Cas9/gRNA system, but also provided a new method and ideas for further gene function. Most importantly, it will provide a technology model for further study the molecular regulation mechanism of Stra8 gene during the differentiating process from embryonic stem cells into male germ cells in vitro.
